# A convenient, soil‐free method for the production of root nodules in soybean to study the effects of exogenous additives

**DOI:** 10.1002/pld3.135

**Published:** 2019-04-15

**Authors:** Swarup Roy Choudhury, Sarah M. Johns, Sona Pandey

**Affiliations:** ^1^ Donald Danforth Plant Science Center St. Louis Missouri

**Keywords:** ABA, auxins, BR, cytokinins, GA, in vitro nodule development, JA, nodulation, phytohormones, soybean

## Abstract

Legumes develop root nodules that harbor endosymbiotic bacteria, rhizobia. These rhizobia convert nitrogen to ammonia by biological nitrogen fixation. A thorough understanding of the biological nitrogen fixation in legumes and its regulation is key to develop sustainable agriculture. It is well known that plant hormones affect nodule formation; however, most studies are limited to model legumes due to their suitability for in vitro, plate‐based assays. Specifically, it is almost impossible to measure the effects of exogenous hormones or other additives during nodule development in crop legumes such as soybean as they have huge root system in soil. To circumvent this issue, the present research develops suitable media and growth conditions for efficient nodule development under in vitro, soil‐free conditions in an important legume crop, soybean. Moreover, we also evaluate the effects of all major phytohormones on soybean nodule development under identical growing conditions. Phytohormones such as abscisic acid (ABA) and jasmonic acid (JA) had an overall inhibitory effect and those such as gibberellic acid (GA) or brassinosteroids (BRs) had an overall positive effect on nodule formation. This versatile, inexpensive, scalable, and simple protocol provides several advantages over previously established methods. It is extremely time‐ and resource‐efficient, does not require special training or equipment, and produces highly reproducible results. The approach is expandable to other large legumes as well as for other exogenous additives.

## 1 INTRODUCTION

Nitrogen is an essential element for plant growth, development and productivity. Improving the nitrogen availability to plants results in significant increases in crop yields. Although present in huge quantities in the atmosphere (78% of earth's atmosphere), this nitrogen is not available to plants, unless fixed by biological nitrogen fixation (BNF). BNF happens by the activity of specialized groups of bacteria called rhizobia, which exists as symbionts with the roots of leguminous plants in specialized structures called root nodules. Root nodule formation is a sophisticated process that requires strict synchronization of bacterial infection and growth as well as plant organogenesis and nodule development. The successful interactions between the host plant and the soil bacteria of *Rhizobium* spp. begin with the secretion of flavonoids from plant roots. In response, the rhizobia produce lipochito‐oligosaccharides, known as nodulation factors or nod factors (NFs). The secreted NF from symbiotically compatible rhizobia directly bind with and activate the nod factor receptors (NFRs) of plants, which are LysM (Lysine motif)‐containing receptor like kinases (Limpens et al., 2003; Madsen et al., 2003; Radutoiu et al., 2003). NFR activation induces root hair deformation, curling, and consequently entrapment of bacteria in those root hairs. The entrapped bacteria form infection threads, which enters in the root hair cells and elongates from the root hair tips to the inner cells to initiate early infection. Additionally, active NFRs stimulate downstream signaling pathways through nuclear Ca^2+^ oscillations and Ca^2+^ spiking to begin nodule organogenesis from the cortical cells (Gleason et al., 2006; Tirichine et al., 2006). All these signaling and organogenesis events are considerably affected by the hormonal balance in plants (Ryu, Cho, Choi, & Hwang, 2012).

Phytohormones both positively and negatively regulate nodulation and nitrogen fixation in legumes. The positive effects of plant hormones auxins and cytokinins in nodule development have been established for a long time. Auxins are a prerequisite during the development and differentiation of nodule primordia and the formation of the vasculature within the nodules (Kohlen, Ng, Deinum, & Mathesius, 2018; Takanashi, Sugiyama, & Yazaki, 2011; Thimann, 1936). Similarly, cytokinins are responsible for the cortical cell division, differentiation, and nodule organogenesis (Frugier, Kosuta, Murray, Crespi, & Szczyglowski, 2008; Gonzalez‐Rizzo, Crespi, & Frugier, 2006; Reid et al., 2017). In addition to auxins and cytokinins, gibberellins (gibberellic acid, GA) are also involved during regulation of nodulation likely via their cross talk with cytokinin signaling pathways (Maekawa et al., 2009). Conversely, stress‐related hormones such as jasmonic acid (JA), salicylic acid (SA), and abscisic acid (ABA) typically reduce nodulation by disrupting NF‐induced Ca^2+^ spiking and downstream signaling pathways (Martinez‐Abarca et al., 1998; Nakagawa & Kawaguchi, 2006; Phillips, 1971).

Nodulation is an energy‐demanding process, therefore to control the number of nodules, legumes have evolved a systemic auto‐regulation of nodulation (AON) as well as local hormonal inhibition of nodulation, which are considered the negative feedback systems. The molecular mechanism of AON has been actively investigated using different supernodulation mutants, such as *hyper nodulation and aberrant root 1 (har1), super numeric nodules 1 (sunn)*, and *nodule autoregulation receptor kinase (nark)* in *Lotus japonicus, Medicago truncatula*, and *Glycine max*, respectively (Ferguson et al., 2010; Krusell et al., 2002; Nishimura et al., 2002; Oka‐Kira & Kawaguchi, 2006; Oka‐Kira et al., 2005; Searle et al., 2003). Numerous studies suggest that auxin, JA, and brassinosteroids (BRs) modulate AON signaling pathways (Kinkema & Gresshoff, 2008; Nakagawa & Kawaguchi, 2006; Oka‐Kira et al., 2005; Terakado, Yoneyama, & Fujihara, 2006) whereas ABA, JA, ethylene, and SA appear to act as during local inhibitory regulation of nodulation (Biswas, Chan, & Gresshoff, 2009; Ding et al., 2008; Oldroyd, Engstrom, & Long, 2001; Penmetsa & Cook, 1997; Sun et al., 2006). Coordinated action of the hormone levels and signaling controls nodule organogenesis and mature nodule development.

Previous studies on the hormonal control of nodulation are based on physiological approaches using a variety of leguminous species and exogenous application of phytohormones to study their effect on nodule formation. For example, exogenous application of cytokinins and auxins to pea root cortical explants induced cell proliferation required for root nodule formation (Libbenga, van Iren, Bogers, & Schraag‐Lamers, 1973). On the other hand, exogenous ABA reduced the number of root nodules by inhibiting the cortical cell divisions during nodule organogenesis (Phillips, 1971). GA, an important growth regulator, also modulates root nodule formation in legumes by exogenous application (Maekawa et al., 2009). SA, a key molecule in plant disease resistance, was shown to inhibit the indeterminate nodules of *Vicia sativa*, but not the determinate nodules of *Lotus japonicus* after exogenous application (van Spronsen et al., 2003). Although each hormone had a characteristic physiological effect, it was evident that different hormones may also follow additive, synergistic, or antagonistic interactions to regulate nodule formation.

The availability of excellent mutant populations in plants such as *L. japonicus* and *M. truncatula* provided genetic evidence for the effect of hormones on nodule formation. These plants serve as useful models due to their modest genome sizes, short seed‐to‐seed generation time, high plant transformation efficiency, and the formation of a restricted number of root nodules. These traits are also useful for performing highly controlled in vitro assays with multiple exogenous additives, which has led to several key discoveries (Bensmihen, 2015; Maekawa et al., 2009; Nakagawa & Kawaguchi, 2006; Stacey, McAlvin, Kim, Olivares, & Soto, 2006; Sun et al., 2006). Conversely, it is difficult to perform similar assays with exogenous additives in crop legumes such as soybean, due to their large stature, long life cycle, the formation of a huge number of root nodules, and the requirement of soil for nodule formation. To circumvent these problems, split‐root system was used in soybean to study the effects of various exogenous variable during rhizobia‐legume symbioses (Chaillou, Rideout, Raper, & Morot‐Gaudry, 1994; Gil‐Quintana et al., 2013; Lin, Gresshoff, & Ferguson, 2012; Singleton & Bohlool, 1984). Most recently, a new split‐root system was developed for continuous monitoring of soybean roots throughout the whole experiment after rhizobial infection (Hidalgo, Ruiz‐Sainz, & Vinardell, 2018). Although these protocols are useful, it is still technically challenging to do some of these experiments and is difficult to apply to a large plant population.

The goals of this study were to identify suitable media and growth conditions for efficient nodule development under in vitro conditions in soybean *G. max*, which is an important crop but not amenable to the standard plate‐based assays used to study nodule formation in *Medicago* or *Lotus* sp. In addition, the effect of each of the major phytohormones was analyzed on nodule development under a standard set of conditions. The results presented in the following sections describe a set of optimum growth and treatment conditions for soil‐free soybean nodulation and effects of phytohormones on it, which will be useful for the community at large.

## 2 MATERIALS

1. Soybean seeds (*William 82*)
2. Pots (2‐gallon) filled with soilrite (BM 7, 35% Bark mix, Berger, Saint‐Modeste, QC, Canada)
3. Rock wool, cubic size (Hummert International, Earth City, MO, USA)
4. Petri dishes (100 × 15 mm) (Corning^™^ Falcon^™^, Fisher Scientific, USA)
5. Trays (2 square feet) (T.O. Plastics, Inc., Clearwater, MN, USA)
6. Pot (85 × 85 mm) (T.O. Plastics, Inc.)
7. Soilrite (Vermiculite:Perlite:Sand in 3:1:1 ratio) containing pots (Therm‐O‐Rock East Inc, New Eagle, PA, USA)
8. Beakers (250 ml)
9. Germination paper (Anchor Paper Company, St Paul, MN, USA)
10. Light chamber under 16 h light/8 h dark conditions (100 μmol m^−2^ s^−1^) with 50% humidity and 25°C (day/night) temperature
11. Greenhouse with 50% humidity and 31/22°C (day/night) temperature.
12. Nitrogen‐free nutrient solution (1000× stock solution, pH 6.5, sterile)

**Table:** 

Macronutrient	
KSO_4_	(87.135 gm/L)
KH_2_PO_4_	(68.045 gm/L)
CaCl_2_, 2H_2_O	(147.01 gm/L)
MgSO_4_, 7H_2_O	(123.24 gm/L)
Fe‐EDTA	
EDTA	(372.24 gm/L)
FeSO_4_, 7H_2_O	(278.02 gm/L)
Micronutrient	
ZnSO_4_, 7H_2_O	(0.143 gm/L)
CuSO_4_, 5H_2_O	(0.030 gm/L)
MnSO_4_, H_2_O	(0.845 gm/L)
H_3_BO_3_	(1.855 gm/L)
(NH_4_)_6_Mo_7_O_24_·4H_2_O	(0.099 gm/L)
Co(NO_3_)_2_	(0.003 gm/L)
NiSO_4_	(0.026 gm/L)
**13.** Vincent's rich medium (per liter)	
K_2_HPO_4_	(0.5 gm)
NaCl	(0.1 gm)
MgSO_4_, 7H_2_O	(0.2 gm)
Yeast Extract	(0.4 gm)
Mannitol	(10.0 gm)
pH 6.8

14 Rhizobium strain: *Agrobacterium rhizogenes* strain K599 (USDA136)
15 Plant hormones:
  a (+/−) Abscisic acid (ABA), (Caisson Labs, Smithfield, UT, USA)
  b Indole‐3‐Acetic Acid (IAA), (Caisson Labs)
  c 6‐Benzylaminopurine (BAP), (Caisson Labs)
  d Epibrassinolide ≥ 85% (BR), (Sigma‐Aldrich, Saint Louis, MO, USA)
  e Gibberellic Acid (GA_3_), (Caisson Labs)
  f Methyl jasmonate, 95% (JA), (Sigma‐Aldrich)
  g Methyl salicylate *ReagentPlus* ^®^, ≥99% (GC) (SA), (Sigma‐Aldrich)

## 3 METHODS

### 3.1 Plant growth

**Figure 1:**
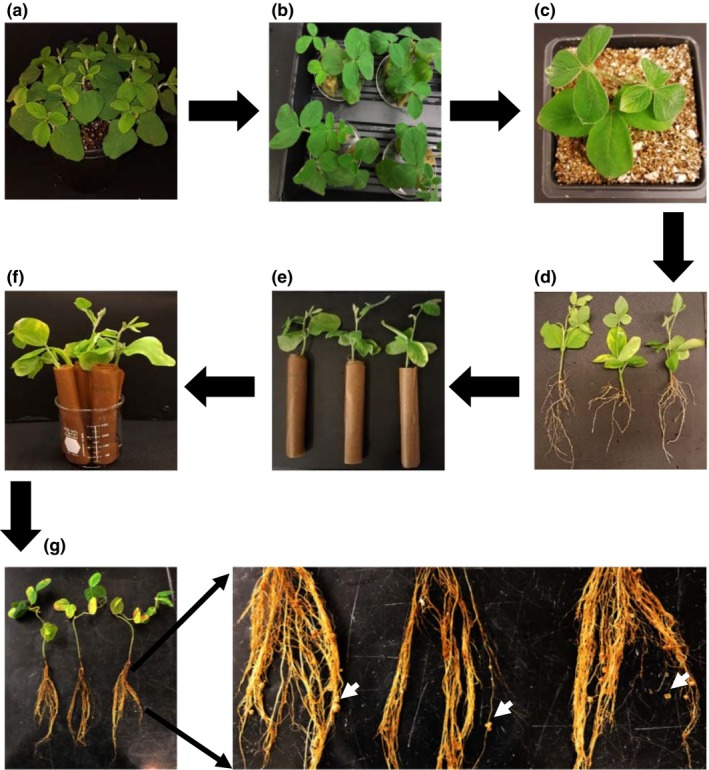
Experimental flowchart describing the details of soil‐free nodule development on hairy roots of soybean. (a) Two‐week‐old plants grown on soil were used as the source of trifoliate leaves. (b) The trifoliate leaves were removed the plants and inserted in the middle of sterilized rock wool cubes for the development of hairy roots for 2 weeks. (c) After 2 weeks, plants with hairy roots were transferred to the pots with soilrite and allowed to grow for 1 week followed by infection with rhizobium. (d) Post‐infected plants (72 h after rhizobium infection) were removed from the pot. (e) Plants were wrapped with pre‐wet germination paper in rolls. One plant was placed per roll. (f) Six plants were kept in each beaker containing nitrogen‐free nutrient solution. (g) Nodule number was counted after 4 weeks of further growth

Soybean seeds were grown in the greenhouse in two‐gallon pots filled with soilrite (BM 7 35%) for 12–14 days at (25/24°C (day/night) temperature, 40%–95% humidity, and ∼300–450 μmol/m^2^/s light intensity depending on the weather and time of the day. Twenty‐five to thirty seeds were grown in each pot, with equal spacing (Figure 1a). There should be no overlap of the seeds. The spacing is important for proper growth of the seedlings.

### 3.2 Hairy root formation

Hairy roots were developed as per our previously established protocols (Roy Choudhury & Pandey, 2013). Briefly, three to four sterilized rock wool cubes were placed in one Petri dish and a hole was created in the middle of the rock wool by using 1 ml pipette tips. Six milliliter nitrogen‐free nutrient solution was applied in the hole of each rock wool cube. The trifoliate leaf from the upper part of a 2 weeks old soybean plant was cut using a razor blade and put in the hole of the rock wool cube (Figure 1b). Eight Petri dishes (~30 trifoliate) could be placed in each two square feet tray.

The trays were covered with a plastic cover and kept in the light chamber with the light intensity 100 μmol m^−2^ s^−1^. Plants were grown for 2 weeks for generation of hairy roots. If needed, the nitrogen‐free nutrient solution was added in the rock wool cubes. After 2 weeks, the rock wool cubes were carefully removed and the plants with hairy roots were transferred to the 85 mm × 85 mm pots containing soilrite (vermiculite:perlite:sand at a 3:1:1 ratio). The plants were grown for one more week in the greenhouse (Figure 1c).

### 3.3 Rhizobium treatment

The appropriate *Rhizobium* strain was cultured in Vincent's rich medium containing chloramphenicol (20 μg/ml) for 3–4 days. The culture was spun down and resuspended in equal volume of nitrogen‐free nutrient solution, with the final concentration adjusted to 0.08 at OD_600_. Ten milliters of rhizobium culture was poured surrounding the roots in each pot and plants were grown for 2–4 days in the green house.

### 3.4 Hormone treatment

After rhizobium inoculation, plants were removed from the pot at specific time points (Figure 1d), wrapped in a roll between two sheets of germination paper pre‐wet with the nitrogen‐free nutrient solution. Six plants (in individual rolls) were kept in one 250 ml beaker containing 100 ml of nitrogen‐free nutrient solution (Figure 1e). Specific hormones were added to each beaker one time every week, immediately after transfer of the plants to the media. The 100 ml nitrogen‐free nutrient solution level was maintained by monitoring the solution level on alternate days and adding media as required. Control and treatment plants were treated similarly to avoid any non‐biological variation. Multiple replicates were performed for each treatment. Nodule numbers were counted after 4 weeks of growth (Figure 1f). The nodules were divided into small (<0.5 mm in diameter), medium (0.5–2 mm in diameter), and large (>0.2 mm in diameter) categories.

### 3.5 Statistical analysis

All experiments were repeated two times independently and the data were averaged. Each replicate consisted of 24 plants per treatment per concentration. Statistical significance of results was calculated using Student's *t* test with a *p*‐value threshold of less than 0.05.

## 4 RESULTS AND DISCUSSION

### 4.1 Optimization of soil‐free nodule development in soybean

**Figure 2:**
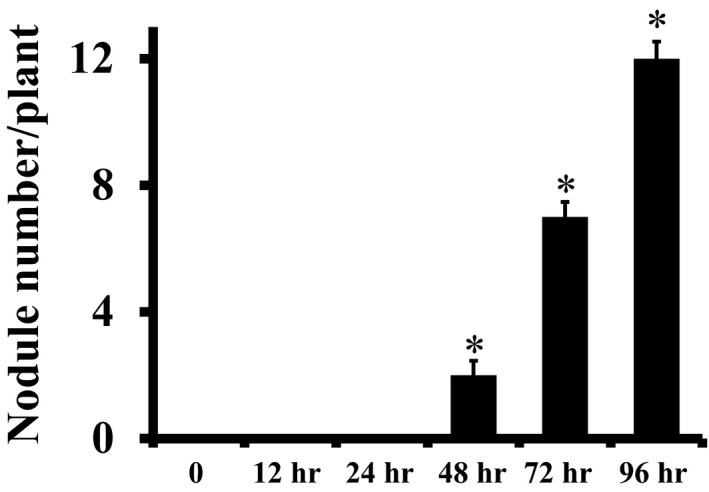
Optimization of nodule development time after infection. Rhizobia‐infected soybean hairy roots were transferred to pre‐wet germination paper rolls at different time points. Nodule number was counted 4 weeks after infection. All experiments were repeated two times independently and data were averaged. Each replicate consisted of 24 plants. Asterisks denote significant difference, *p < 0.5, Student's *t* test

Symbiotic nitrogen fixation in nodules plays a key role in the maintenance of soybean seed production. While in plants such as *Medicago*, their small stature allows for growth under in vitro conditions on sterile media plates, the large size of soybean plants precludes such a possibility. The study of soybean nodule development in soil by using different additives is relatively hard, inaccurate, and expensive. To overcome these issues, soil‐free nodule production under in vitro condition is a suitable choice. We optimized a method where the nodules were allowed to develop in germination papers, after initial infection in soil (Figure 1). Briefly, trifoliate leaves with a stalk were cut from 2‐week‐old plants grown on soil and inserted in the middle of sterilized rock wool cubes (Govindarajulu et al., 2009; Libault et al., 2009). The rock wool in cubes causes abrasion, which promotes the development of hairy roots, which are well developed after 2 weeks of growth. After this time plants with hairy roots were transferred to small pots (85 mm × 85 mm) containing soilrite (vermiculite:perlite:sand in a 3:1:1 ratio) and allowed to grow for 1 week. For nodule development, plants in pots were infected with compatible rhizobium strains. Infection of plants in soilrite was critical, because infecting plants in germination papers by direct application of rhizobia would result in uncontrolled bacterial infection on the paper itself. Plants were transferred 72 h post‐infection between the two sheets of pre‐wet germination paper and were arranged in rolls. One plant was placed per roll and each 250 ml beaker can accommodate six rolls. The plants were allowed to grow in nitrogen‐free nutrient solution and nodule number was counted after 4 weeks of further growth. For nodule development in soilrite, the plants can continue to grow in pots after infection, but will require one ½ gallon pot per plant. Achieving uniform root growth and nodule formation in soybean under in vitro conditions has been a challenge as it shows enormous inconsistencies in nodule numbers. In our experiments, the time of bacterial infection and the duration of subsequent growth of plants in soilrite were critical for efficient nodule development at later stages. To optimize the conditions for consistent, reproducible results, we transferred plants from soilrite to germination paper rolls at different time points after rhizobium infection and counted the nodules after 4 weeks of growth. Plant relocation time after rhizobium infection had a huge effect on nodule formation (Figure 2). No nodules were formed if we transferred the plants from 0 to 24 h after rhizobium infection, whereas ~2, 8, and 12 nodules on average were formed per plant if the plants were transferred 48, 72, and 96 h after rhizobium infection, respectively. Based on these results, all through our experiments, we have transferred the plants from soilrite to germination paper rolls 72 h post‐infection as it generates a reasonable number of nodules needed for any comparative analysis.

We are cognizant of the fact that the need to grow roots in soilrite for up to 72 h post‐infection excludes the possibility of assaying the effects of various additives during the early stages of infection such as on root hair curling, or infection thread formation. The method described in this research is therefore suitable for the study of additives at the later stages of nodule development only. However, as detailed in the next sections, it does result in reproducible and consistent effects of exogenous additives on nodule developments and is extremely resource‐ and cost‐effective.

### 4.2 Optimization of hormone treatment time

For this study, we focused on assaying the effects of various plant hormones as exogenous additives, as these have a significant influence not only on nodule initiation but also on organogenesis. Similar assays can be optimized for other additives such as salt, mannitol (for osmotic stress) etc. as needed. The hormone treatments were started when the plants were transferred from soilrite, and germination paper rolls containing plants were transferred to either control or treatment media. An obvious effect on nodule development was observed for each of the plant hormones.

### 4.3 Effect of ABA on root nodule development

Abscisic acid regulates multiple aspects of rhizobia‐legume symbiosis (Bano & Harper, 2002; Bano, Harper, Auge, & Neuman, 2002; Cho & Harper, 1993; Phillips, 1971). Because the addition of exogenous ABA causes a marked increase in the endogenous ABA levels in plants, the application of ABA to media in which plants are growing is a suitable system to study its effect on nodulation. In *L. japonicus*, the number of root nodules is reduced at higher ABA concentrations, and increased in the presence of abamine (which causes lower ABA concentrations) suggesting its inhibitory role during nodule development (Suzuki et al., 2004). In soybean, exogenous ABA decreased nodule number in both the wild‐type and a supernodulation mutant (Bano & Harper, 2002; Bano et al., 2002; Cho & Harper, 1993).

**Figure 3:**
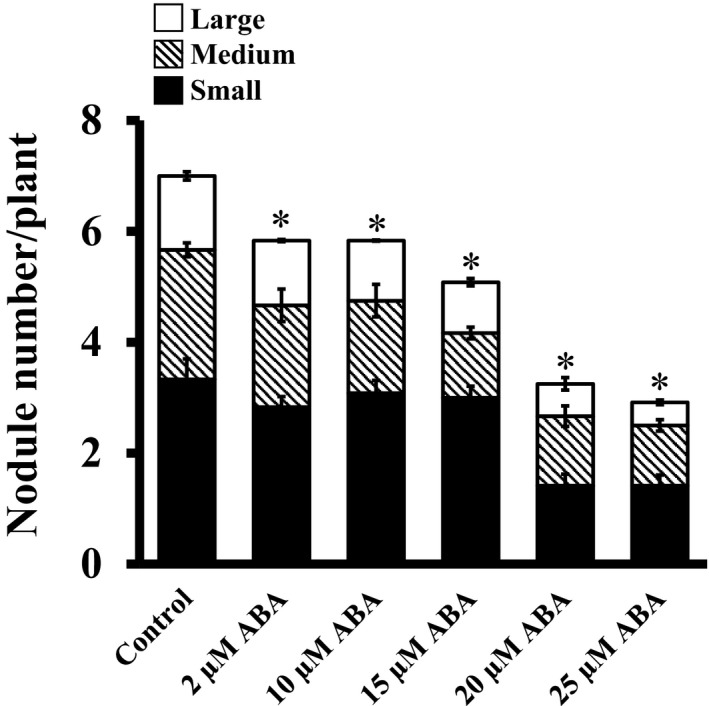
Effect of exogenous ABA on nodule formation. Rhizobia‐infected soybean hairy roots were treated with different concentrations of ABA when transferred to the nitrogen‐free media. ABA concentration was maintained throughout the experiment (4 weeks). Nodule number was counted 4 weeks after infection. All experiments were repeated two times independently and data were averaged. Each replicate consisted of 24 plants. Asterisks denote significant difference, *p < 0.5, Student's *t* test

We evaluated the effect of ABA on soybean nodulation in a concentration‐dependent manner using ABA concentrations ranging from 2 to 25 μM; concentrations lower than 2 μM were ineffective under our growth conditions. An equimolar amount of ethanol was used as a control in all assays. Nodule formation showed extreme sensitivity to exogenously added ABA. On average, seven nodules were formed per plants after 4 weeks of growth under control conditions, which was reduced by 15%–30% in presence of 2–15 μM ABA (Figure 3). It was noticeable that plants treated with ABA had fewer large (>2 mm in diameter) and medium (0.5–2 mm in diameter) compared to the control plants but a comparable number of small nodules (<0.5 mm in diameter) were produced (Table S1). Concentrations higher than 15 μM severely affected nodule formation, with the nodule numbers reduced by more than 60% per plant in the presence of 20–25 μM ABA. At these higher concentrations, all types of nodules (large, medium, and small) were significantly affected. These data confirm the inhibitory effects of exogenous ABA on soybean nodule formation, and by extension of other abiotic stresses, which cause an increase in ABA concentrations in planta.

### 4.4 Effect of auxin (IAA) and cytokinin (BAP) on nodule development

Several studies have demonstrated that auxin and cytokinin intricately control root nodule formation. IAA (indole‐3‐acetic acid), a native auxin in plants, derived from the phenylpropanoid biosynthetic pathway is a key member of the auxin family. The root nodules have a higher IAA content than uninfected root tissues, suggesting a role for IAA in nodule development (Thimann, 1936). The higher IAA content of infected roots promotes root cells to undergo cell division, elongation, differentiation, and vascular bundle formation to develop nodules (Boivin, Fonouni‐Farde, & Frugier, 2016; Kuppusamy et al., 2009; Nagata & Suzuki, 2014; Suzaki et al., 2012). Moreover, rhizobia alter the root auxin balance, which is a prerequisite for nodule formation (Boot, van Brussel, Tak, Spaink, & Kijne, 1999; Hirsch, Bhuvaneswari, Torrey, & Bisseling, 1989; Mathesius et al., 1998; Pacios‐Bras et al., 2003; Wasson, Pellerone, & Mathesius, 2006). Besides auxin content, rhizobia‐legume symbiosis is also regulated by shoot‐to‐root auxin transport, which is an active transport process involving auxin efflux protein complexes (van Noorden, Ross, Reid, Rolfe, & Mathesius, 2006). Overall, it is well established that the changes in auxin accumulation and transport, both are essential for lateral root development, nodule primordium activation, and nodule organogenesis (Mathesius et al., 1998; Suzaki et al., 2012; Takanashi et al., 2011).

**Figure 4:**
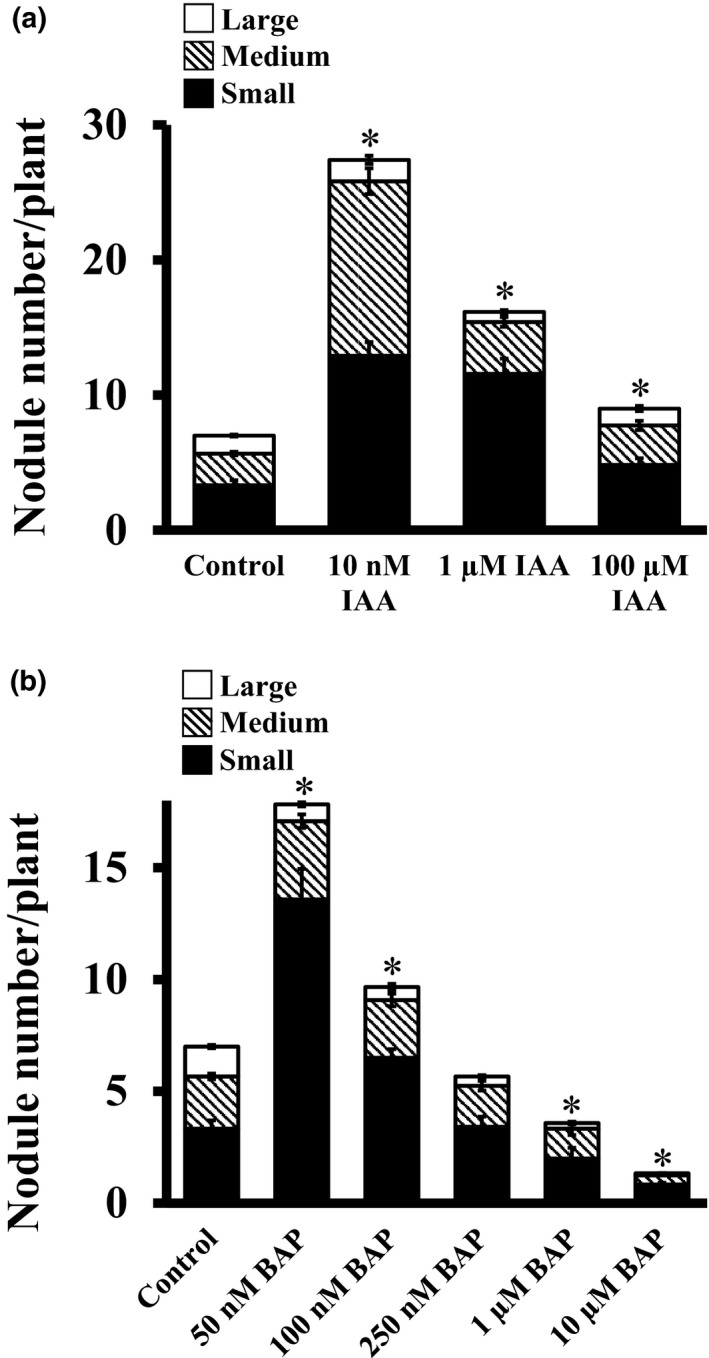
Effect of exogenous auxin (IAA) and cytokinin (BAP) on nodule formation in soybean. Rhizobia‐infected soybean hairy roots were treated with different concentrations of (a) IAA and (b) BAP, when transferred to the nitrogen‐free media. The hormone concentrations were maintained throughout the experiment (4 weeks). Nodule number was counted 4 weeks after infection. All experiments were repeated two times independently and data were averaged. Each replicate consisted of 24 plants. Asterisks denote significant difference, *p < 0.5, Student's *t* test

To test the effect of exogenous auxins on soybean nodule formation, plants were treated with different IAA concentrations ranging from 10 nM to 100 μM. IAA at 10 nM caused the most obvious phenotypic differences in the nodule number, although all treatment conditions resulted in higher nodule number per plant. Approximately four times more nodules were formed per plant at 10 nM IAA compared to control plants with a huge increase in the number of small‐ and medium‐size nodules (Figure 4a). Two times more nodules were formed compared to control conditions in the presence of 1 μM IAA, whereas a modest increase in nodule numbers was observed in the presence of 100 μM IAA in the media. These results indicate that even at a very high concentration, auxin still has a limited but positive effect on nodule formation. Furthermore, 10 nM exogenous auxin is an optimal concentration for increased nodule numbers in soybean.

Extensive research on *L. japonicus* and *M. truncatula* have demonstrated that cytokinins (CKs) are key players in the regulation of rhizobium infection and nodule development (Frugier et al., 2008; Gonzalez‐Rizzo et al., 2006; Tirichine et al., 2007). The activation of NF signaling pathway rapidly induces CK accumulation and response in hairy roots (Buhian & Bensmihen, 2018; Gamas, Brault, Jardinaud, & Frugier, 2017; Murray et al., 2007). The exogenous application of CKs also promotes cortical cell divisions and the expression of early nodulation markers in different legumes (Bauer, Ratet, Crespi, Schultze, & Kondorosi, 1996; Jimenez‐Zurdo, Frugier, Crespi, & Kondorosi, 2000; Mathesius, Weinman, Rolfe, & Djordjevic, 2000; Murray et al., 2007; Tirichine et al., 2007). To study the effect of exogenous applications of cytokinins on soybean nodulation, we included different concentrations of BAP (6‐Benzylaminopurine) (50 nM, 100 nM, 250 nM, 1 μM, and 10 μM) in the media when the plants were transferred from soilrite to germination paper rolls. We detected a clear, concentration‐dependent effect of BAP on nodulation. Approximately 2.5 times more nodules were formed in the presence of 50 nM BAP, whereas a modest increase was observed in the presence of 100 M BAP, compared to the control conditions (Figure 4b). Interestingly, BAP concentrations higher than 250 nM were inhibitory and lead to the development of fewer nodules compared to control media grown plants. For example, ~80% less nodule developed in the presence of 10 μM BAP. These data suggest that nodule formation in soybean strongly regulated by precisely controlled CKs level.

### 4.5 Effect of GA and BR on root nodule development

**Figure 5:**
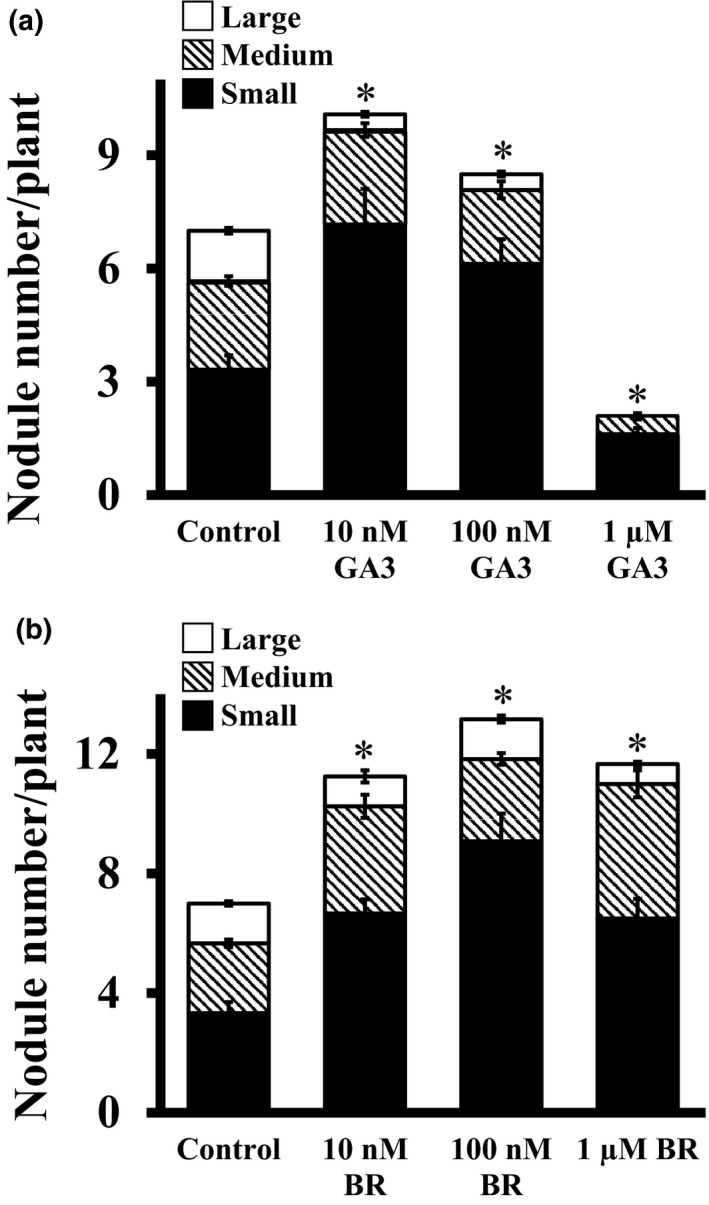
Effect of gibberellic acid (GA_3_) and brassinosteroids (brassinolide, BR) on nodule formation in soybean. Rhizobia‐infected soybean hairy roots were treated with different concentrations of (a) GA_3_ and (b) Brassinolide, when transferred to the nitrogen‐free media. The hormone concentrations were maintained throughout the experiment (4 weeks). Nodule number was counted 4 weeks after infection. All experiments were repeated two times independently and data were averaged. Each replicate consisted of 24 plants. Asterisks denote significant difference, *p < 0.5, Student's *t* test

Gibberellic acid is one of the vital growth regulators in higher plants. Several studies have highlighted the involvement of GA in the regulation of the rhizobium‐legume symbiotic interaction (Ferguson, Ross, & Reid, 2005; Lievens et al., 2005; Maekawa et al., 2009; McAdam, Reid, & Foo, 2018). Interestingly, both positive and negative effects of GA have been reported in previous studies. For examples, one study reported the inhibitory effects of an exogenous application of potassium gibberellate on nodule formation in *Phaseolus vulgaris* (Thurber, Douglas, & Galston, 1958). On the other hand, nodules aborted in pea mutant (*na‐1*) which was deficient in GA_3_, but were re‐established by the application of exogenous GA_3_ (Ferguson et al., 2005) implying requirement of GA for nodule formation. This discrepancy in the results could be either the concentration of GA used or the specific plant species used in the assays. To assess the effect of GA_3_ on soybean nodule formation, plants post‐infection were treated with different GA_3_ concentrations (10 nM to 1 μM) and compared with control media with no added GA_3._ Plants treated with 10 nM and 100 nM GA_3_ showed considerably increased nodule formation with ~40% and ~20% more nodules, respectively. Interestingly, most of the nodules in these treatments were small (Table S1). However, similar to what was observed for cytokinins, at higher concentrations (1 μM) of GA_3_, the nodule formation was severely affected and ~70% fewer nodules were formed on treated roots compared with control media (Figure 5a). These data suggest that the reported discrepancies in the previous publications could be due to the different GA concentrations used in different assays. These observations suggest that the endogenous GA concentration in plants is tightly regulated to achieve effective nodulation.

Brassinosteroids play pivotal roles during many aspects of plant growth and development (Clouse & Sasse, 1998; Wei & Li, 2016). BRs affect both cell proliferation and cell elongation to control shoot and root lengths and hypocotyl growth in plants (Gonzalez‐Garcia et al., 2011; Tong et al., 2014). Although the effects of BRs are well documented on root and shoot growth, their effects on nodule development in different legumes are not as well described. In one example, BRs affected nodule development by reducing lateral root numbers in pea as is evident from the BR synthesis mutants *lk* and *lkb* and the BR response mutant *lka* (Ferguson et al., 2005). To test the effect of BRs on soybean nodule development, plants were treated with media containing different brassinolide (active BR) concentrations ranging from 10 nM to 1 μM. In comparison to plant grown on control media, a concentration‐dependent increase in nodule numbers was observed in plants grown on BR containing media. Highest nodule numbers were seen in response to 100 nM brassinolide, where ∼85% more nodules were present compared to the control media roots (Figure 5b). At lower (10 nM) and higher (1 μM) concentrations of exogenous BL, ∼50% more nodules were formed, suggesting the BRs are positive regulators of nodule formation.

### 4.6 Effect of SA and JA on root nodule development

**Figure 6:**
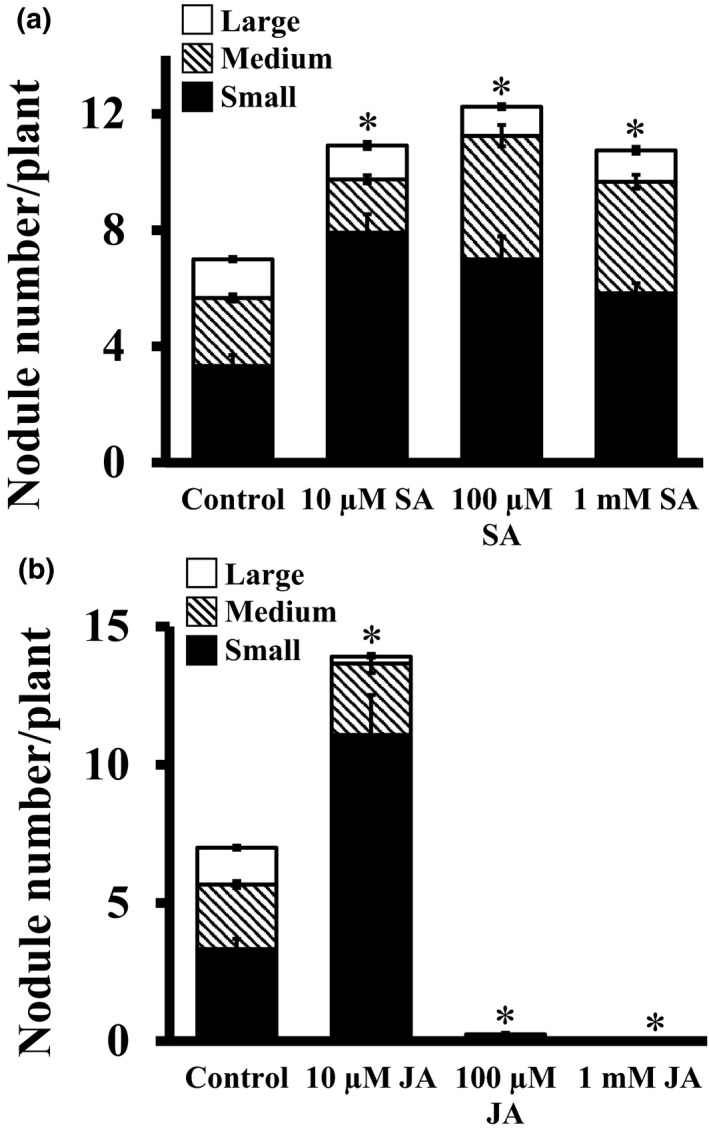
Effect of salicylic acid (SA) and jasmonic acid (JA) on nodule formation in soybean. Rhizobia‐infected soybean hairy roots were treated with different concentrations of (a) SA and (b) JA, when transferred to the nitrogen‐free media. The hormone concentrations were maintained throughout the experiment (4 weeks). Nodule number was counted 4 weeks after infection. All experiments were repeated two times independently and data were averaged. Each replicate consisted of 24 plants. Asterisks denote significant difference, *p < 0.5, Student's *t* test

Salicylic acid strongly affects nodule formation at early stages of nodulation (Sato et al., 2002). The exogenous application of SA resulted in both reduced and delayed nodule formation on *Medicago* roots inoculated with wild‐type *S. meliloti*. Moreover, exogenous SA that inhibited nodulation also strongly reduced the growth of the bacterial symbiont (Martinez‐Abarca et al., 1998). The effect of SA was additionally documented in different legumes. For example, the inhibition of nodule formation after exogenous SA treatment was observed in plants like vetch (*Vicia sativa*), pea (*Pisum sativum*), and white clover (*Trifolium repens*) (van Spronsen et al., 2003). This study shows that the exogenous SA inhibits indeterminate but not determinate nodulation (van Spronsen et al., 2003), which is inconsistent with two other previous reports (Lian, Zhou, Miransari, & Smith, 2000; Sato et al., 2002), where they showed that exogenous application of SA to soybean, which forms determinate nodules, reduced nodulation. Another report demonstrated that SA enhances the efficiency of nitrogen fixation and assimilation in *Cicer arietinum* (Hayat, Hayat, Alyemeni, & Ahmad, 2014). To examine the role of exogenous SA in soybean nodulation in our system, we used different concentrations of SA ranging from 10 μM to 1 mM in the exogenous media. All SA‐treated soybean plants exhibited a significantly higher nodule number, ranging from 57% to 76% compared to the control media (Figure 6a). The differences were more prominent in case of higher concentration of SA treatment (100 μM to 1 mM). Our data thus suggest that exogenous SA levels positively regulate nodulation in soybean at these concentrations.

Jasmonic acid negatively regulates plants’ response to the rhizobial bacterial signal, NF (Sun et al., 2006). Primarily, JA inhibits nodule formation by suppressing calcium spiking and the frequency of calcium oscillations to modulate the NF‐induced gene expression (Sun et al., 2006). In addition, even in the shoots, the AON pathway is modulated by JA (Kinkema & Gresshoff, 2008). However, a recent study by suppression of allene oxide cyclase in *M. truncatula*, an enzyme involved in committed step in JA biosynthesis, suggested that jasmonates are not involved in the development and function of root nodules (Zdyb et al., 2011). To examine if exogenous JA has any effect on soybean nodulation, we tested its effects at different concentrations (10 μM, 100 μM, and 1 mM). Surprisingly, at low concentration (10 μM), exogenous JA showed a positive effect and approximately twice as many nodules were formed compared to the control media. However, the nodule number was significantly inhibited (up to 95%) after increasing the JA concentrations from 10 to 100 μM. No nodules were formed in the presence of 1 mM JA (Figure 6b). These data suggest that regulation of nodule formation by JA is complex and is highly dependent on the exogenous concentrations. As hormones such as JA and SA not only modulate the signaling pathways in plants but are also a core part of plant–microbe interaction (Durner, Shah, & Klessig, 1997; Koornneef & Pieterse, 2008; Niki, Mitsuhara, Seo, Ohtsubo, & Ohashi, 1998), it is expected that different concentrations may have altered effects on growth and development versus survival.

## 5 CONCLUSION

Nodule development in legumes directly affects nitrogen fixation efficiency during plant growth. Here, we present a method for determining the effects of ABA, auxin, BAP, GA, BR, SA, and JA on soybean nodulation that is rapid, accurate, technically simple, and requires minimal resources. This method provides several advantages over other methods as these approaches do not require time‐consuming additional steps such as changing solvents and maintaining hormonal concentrations day‐by‐day nor use of large containers, which require large quantities of hormones, space, and tedious handling. Continued manipulations often increases potential technical errors. The method can also be applied to roots coming out of seeds, if generating the transgenic hairy roots is not a requirement. Moreover, the standardization of hormone concentrations and the description of resultant phenotypes will support further targeted studies, and in combination with additional genetic and genomic tools being developed in multiple labs, will greatly increase its use for the study of the effects of exogenous factors affecting nodulation in soybeans as well as in other larger legumes. Similarly, for the study of gene silencing, overexpression or gene editing on nodule development, Agrobacterium cells (*K599*) expressing the appropriate gene constructs can be used in this method by mixing it with a nitrogen‐free nutrient solution, which will result in the generation of transgenic soybean hairy roots.

## AUTHOR CONTRIBUTION

Conceptualization, Sona Pandey; Data curation, Swarup Roy Choudhury and Sarah Johns; Formal analysis, Swarup Roy Choudhury; Funding acquisition, Sona Pandey; Investigation, Swarup Roy Choudhury; Methodology, Swarup Roy Choudhury and Sarah Johns; Project administration, Sona Pandey; Supervision, Sona Pandey; Writing – original draft, Swarup Roy Choudhury; Writing – review & editing, Sona Pandey.
