# Supplementary material for: A convenient, soil‐free method for the production of root nodules in soybean to study the effects of exogenous additives
Source: Plant Direct. 2019 Apr 15;3(4):e00135. doi: 10.1002/pld3.135 (PMC6589526; doi:10.1002/pld3.135)
Supplement: Supplementary file 1 [file PLD3-3-e00135-s001.docx]

**Supplementary Table 1.** Differences in nodule sizes after hormone treatments. Nodules were classified as large (>2 mm in diameter), medium (0.5 to 2 mm in diameter) and small (<0.5 mm in diameter) sizes. Statistical analysis were performed to compare differences in nodule sizes. Asterisks denote signiﬁcant differences (* P < 0.5, Student's *t*-test) when comparing the specific nodule size in control plants versus the treatment plants.

| **Nodules** | 2 µM ABA | 10 µM ABA | 15 µM ABA | 20 µM ABA | 25 µM ABA |
| --- | --- | --- | --- | --- | --- |
| Large |  |  | * | * | * |
| Medium | * | * | * | * | * |
| Small |  |  |  | * | * |
|  | 50 nM BAP | 100 nM BAP | 250 nM BAP | 1 µM BAP | 10 µM BAP |
| Large | * | * | * | * | * |
| Medium | * |  | * | * | * |
| Small | * | * |  | * | * |

|  | 10 nM IAA | 1 µM IAA | 100 µM IAA |
| --- | --- | --- | --- |
| Large |  | * |  |
| Medium | * | * | * |
| Small | * | * | * |

|  | 10 nM GA_3_ | 100 µM GA_3_ | 1 µM GA_3_ |
| --- | --- | --- | --- |
| Large | * | * | * |
| Medium |  | * | * |
| Small | * | * | * |
|  | 10 nM BR | 100 nM BR | 1 µM BR |
| Large | * |  | * |
| Medium | * |  | * |
| Small | * | * | * |
|  | 10 µM SA | 100 µM SA | 1 mM SA |
| Large |  | * | * |
| Medium | * | * | * |
| Small | * | * | * |
|  |  |  |  |
|  | 10 µM JA | 100 µM JA | 1 mM JA |
| Large | * | * | * |
| Medium |  | * | * |
| Small | * | * | * |
